# Polydopamine Nanoparticles Functionalized Electrochemical DNA Aptasensor for Serum Glycated Albumin Detection

**DOI:** 10.3390/ijms232213699

**Published:** 2022-11-08

**Authors:** Pornsuda Maraming, Nang Noon Shean Aye, Patcharee Boonsiri, Sakda Daduang, Onanong Buhome, Jureerut Daduang

**Affiliations:** 1 Centre for Research and Development of Medical Diagnostic Laboratories, Faculty of Associated Medical Sciences, Khon Kaen University, Khon Kaen 40002, Thailand; 2 Department of Biochemistry, Faculty of Medicine, Khon Kaen University, Khon Kaen 40002, Thailand; 3 Division of Pharmacognosy and Toxicology, Faculty of Pharmaceutical Sciences, Khon Kaen University, Khon Kaen 40002, Thailand; 4 Department of Medical Technology, Faculty of Allied Health Sciences, Nakhon Ratchasima College, Nakhon Ratchasima 30000, Thailand

**Keywords:** polydopamine nanoparticles, electrochemical sensor, aptamer, glycated albumin, diabetes mellitus

## Abstract

Polydopamine (PDA) has now been widely applied to electrochemical biosensing because of its excellent biocompatibility, abundant functional groups, and facile preparation. In this study, polydopamine nanoparticles (PDA-NPs)-functionalized electrochemical aptasensor was developed for the rapid, sensitive, and cost-effective detection of glycated albumin (GA), a promising biomarker for glycemic control in diabetic patients. PDA-NPs were synthesized at various pH conditions in Tris buffer. Cyclic voltammetry (CV) of PDA-NPs-coated screen-printed carbon electrodes (SPCEs) revealed that the materials were more conductive when PDA-NPs were synthesized at pH 9.5 and 10.5 than that at pH 8.5. At pH 10.5, the prepared PDA and PDA-aptamer NPs were monodispersed spherical morphology with an average size of 118.0 ± 1.9 and 127.8 ± 2.0 nm, respectively. When CV and electrochemical impedance spectrometry (EIS) were used for the characterization and detection of the electrochemical aptasensor under optimal conditions, the proposed aptasensor exhibited a broad linearity for detection of GA at a clinically relevant range of (1–10,000 µg mL^−1^), provided a low detection limit of 0.40 µg mL^−1^, appreciable reproducibility (less than 10%), and practicality (recoveries 90–104%). In addition, our developed aptasensor presented a great selectivity towards GA, compared to interfering substances commonly present in human serum, such as human serum albumin, urea, glucose, and bilirubin. Furthermore, the evaluation of the aptasensor performance against GA-spiked serum samples showed its probable applicability for clinical use. The developed PDA aptasensor demonstrated excellent sensitivity and selectivity towards GA detection with a simple and facile fabrication process. This proposed technique shows its potential application in GA measurement for improving the screening and management of diabetic patients in the future.

## 1. Introduction

Diabetes mellitus (DM), affecting millions of people worldwide, is a chronic metabolic disorder with heterogenous etiologies resulting from insulin deficiency and/or insulin resistance [1]. Blood glucose level measurement is important in the diagnosis and glycemic monitoring of DM. However, human blood glucose levels are dependent upon food intake and can reflect the glycemic control within the past few hours. Hemoglobin A1c (HbA1c) is recognized as the gold standard for glycemic monitoring. It can provide the average blood glucose level over 2–3 months. Nevertheless, diseases that affect red blood cells (RBCs) including haemoglobinopathy, renal anaemia, haemolytic anaemia, and liver cirrhosis, affect HbA1c measurements [2]. Moreover, due to the long lifespan of RBCs, HbA1c is not suitable for monitoring short-term blood glucose level fluctuations.

Glycated albumin (GA), formed by the non-enzymatic glycation of albumin, is a potential biomarker for monitoring the blood glucose level over the past few weeks. It is specific for the albumin glycation rates and can be used for shorter-term glycemic control than HbA1c and is more accurate than the blood glucose measurement. GA was unaffected by hemoglobin levels or hemoglobinopathies such as anemia, thalassemia, or variant hemoglobin [3]. There are various techniques for measuring GA such as immunoassays, capillary electrophoresis [4], high-performance liquid chromatography, refractive index measurement [5], ion-exchange chromatography, and Raman spectroscopy [6]. However, these techniques are frequently time-consuming, labor-intensive, and require expensive equipment. An electrochemical biosensor is an alternative method to the currently used conventional techniques gaining more attention in recent years [7,8,9]. Its advantages include easy and rapid detection, small volumes of samples, and low-cost instrument [10].

Aptamers are short-sequenced artificial single-strand DNA or RNA that can bind to specific biomolecules [11]. The aptamer can be identified and generated from the oligonucleotide library via a process of Sequential Evolution of Ligands by Exponential Enrichment (SELEX) [12]. Recently, novel techniques using capillary electrophoresis and microfluidic technology significantly enhanced the efficiency of aptamer discovery/selection [13]. Aptamers exhibit complicated three-dimensional structures that are highly selective and able to attach non-covalently to target molecules (small molecules, proteins, lipids, cells, etc.) [14]. Its advantages include low immunogenicity, the ability to be immobilized on various surfaces, high-temperature resistance, similar affinities and specificities compared to antibodies, and better consistency than antibodies [15,16]. Therefore, we developed an aptamer-based biosensor to measure serum GA levels.

Polydopamine (PDA) is a polymerized product from dopamine (3,4-dihydroxyphenethylamine) or other catecholamines under oxidative and basic conditions [17]. PDA was originally used to modify material surfaces that formed thin layers at the nanoscale ranging from a few to about 100 nm (e.g., metal films) [18]. It has good characteristics including biocompatibility, easy steps in preparation, simple functionalization, and thermal stability on practically any surface [19]. However, during PDA film is deposited on the surfaces, the oxidation reaction causes the formation of unwanted aggregation in the solution hindering success in surface modification. This major disadvantage led to PDA production in the form of nanoparticles with colloidal stabilization [18]. PDA nanostructures offer aqueous solubility, good optical and electrical properties, surface modification ability, and photothermal properties offering several applications in biomedical platform and analytical science [20,21]. As for the chemistry of the material surfaces, PDA layers can be modified via various mechanisms such as catechol–metal coordination, electrostatic interactions, π–π interactions, hydrogen, and covalent bonds resulting in the success of surface functionalization [22].

PDA nanoparticles (PDA-NPs) have been applied for the development of the sensor, drug carrier, molecular imaging, gene targeting therapy, and tissue engineering [18]. Moreover, PDA-based films and nanoparticles have demonstrated a promise as sensing materials for a variety of analytes. The number of studies looking at possible uses of this material is still rising quickly. Previous electrochemical studies showed the most common binding molecules for biomarker detection in DM monitoring including antibodies [23], aptamers [24], and enzymes [25]. Until now, PDA-NPs-based electrochemical study has never been explored for serum GA measurement. In this study, a sensitive and selective electrochemical aptasensor was fabricated using the PDA-NPs nanocomposite for label-free GA detection in spiked clinical samples.

## 2. Results and Discussion

### 2.1. Characterization of PDA Nanoparticles

**Figure 1:**
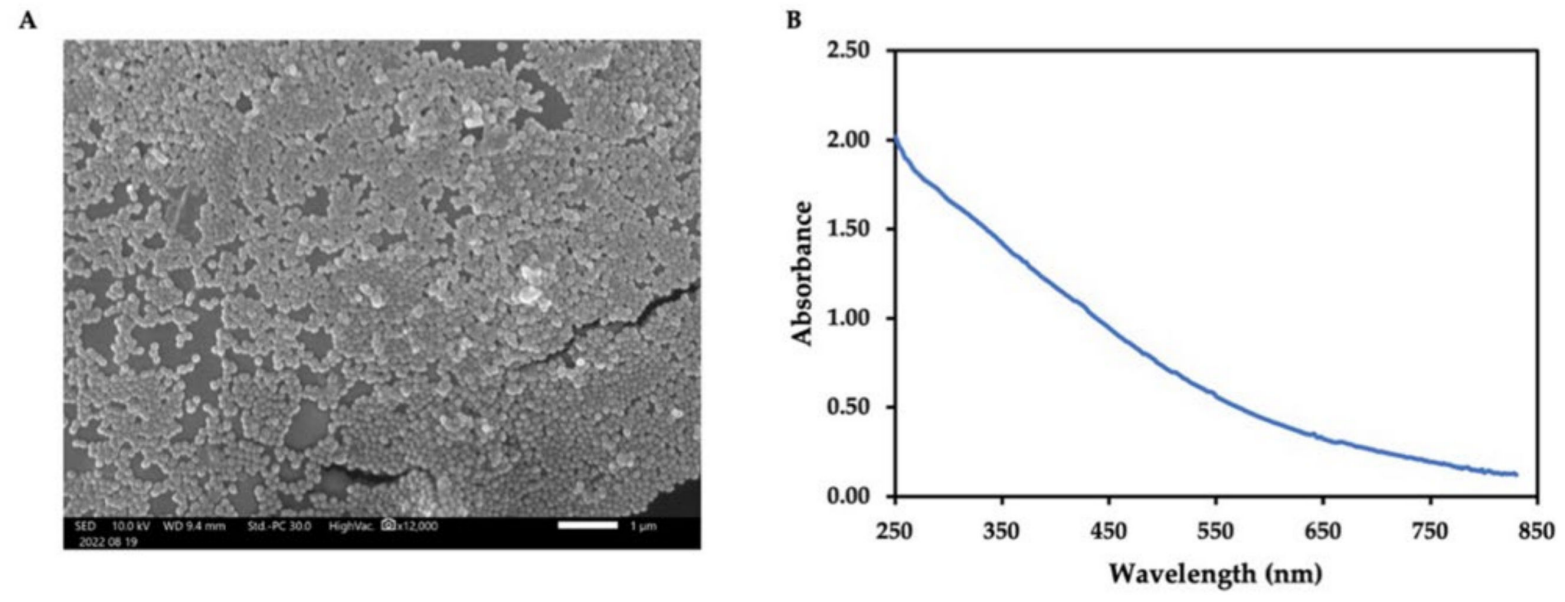
(**A**) SEM image and (**B**) UV-Vis spectrum of synthesized PDA-NPs.

PDA is a biopolymer that is brown to black in color and contains significant amounts of phenol, catechol, and quinone functional groups, which provide a variety of molecular adsorption characteristics [26]. PDA-NPs exhibit the ability to chelate and bind many compounds. In addition to having biocompatibility, good adhesion, and anti-biofouling characteristics, PDA nanostructures are good candidates for biomedical applications [27,28]. There are various methods for synthesizing PDA-NPs. The most commonly used techniques are enzymatic/solution oxidation as well as electro-polymerization [22]. In this study, PDA-NPs were formed by the self-oxidative polymerization of 0.5 mg mL^−1^ dopamine under alkaline conditions. After 20 h of synthesis, PDA-NPs were characterized by scanning electron microscope (SEM), spectrophotometry, and attenuated total reflectance Fourier-transform infrared spectroscopy (FTIR). Morphologically, PDA-NPs observed under SEM were uniform, monodispersed, and spherical shape (see Figure 1A). The UV–Vis spectrum of the synthesized PDA-NPs is shown in Figure 1B. The absorbance pattern decreased with the increase of the wavelength from 250 to 830 nm.

**Figure 2:**
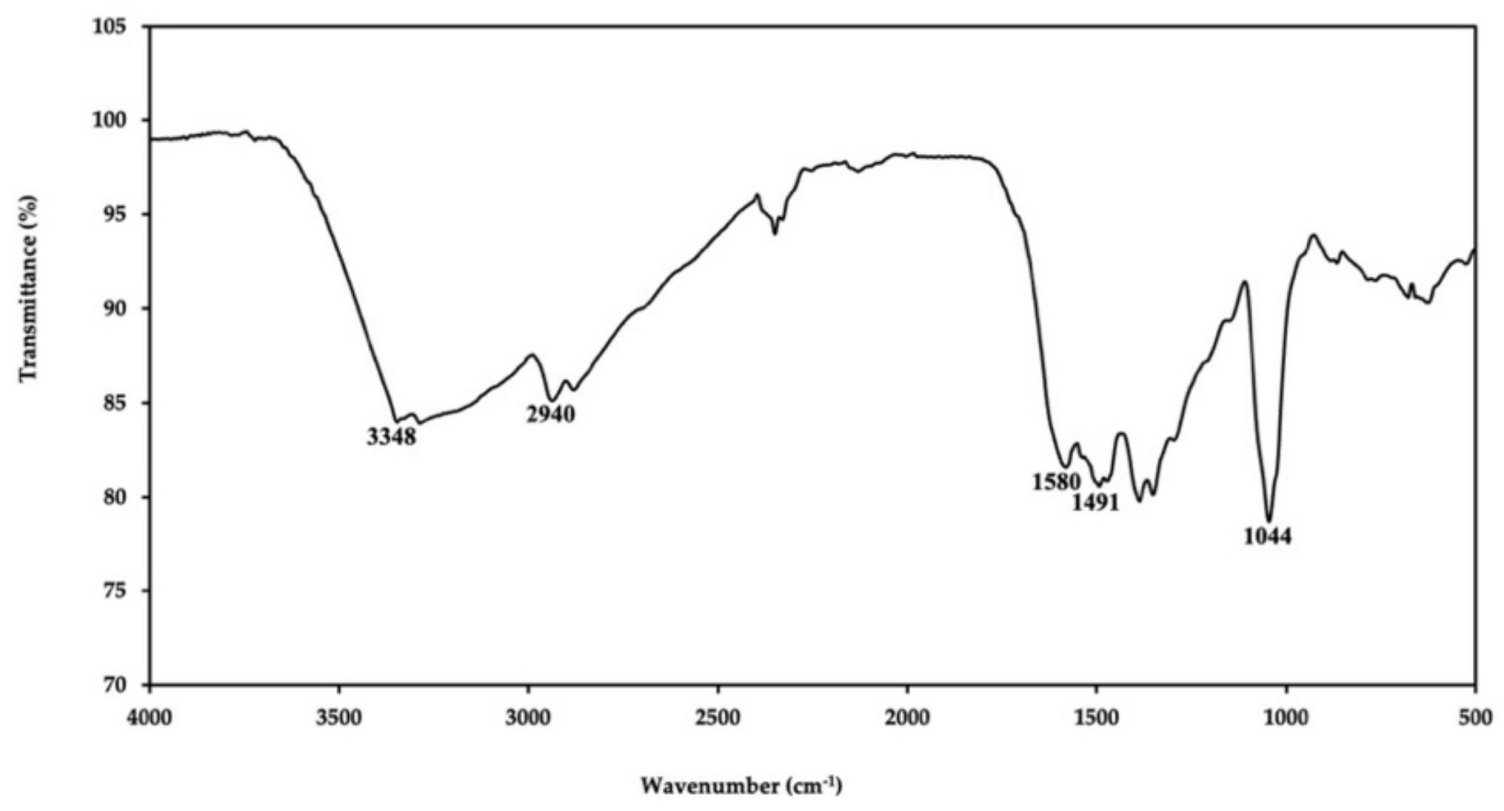
ATR-FTIR spectrum of synthesized PDA-NPs.

The synthesized PDA-NPs were also subjected to FTIR examination in order to analyze further characteristic functional groups of the PDA NPs formation during dopamine oxidation and polymerization in the spectral range between 500 and 4000 cm^−1^ as shown in Figure 2. The peaks at 3348 cm^−1^ and 2940 cm^−1^ were observed in the PDA-NPs spectrum corresponding to O-H stretching vibrations and C–H stretching of the aromatic ring, respectively [28,29,30]. The characteristics of C=C and C–N–C stretching from the indole group of PDA-NPs were found by peaks at 1580 and 1491 cm^−1^, respectively [20,31,32,33]. Moreover, a strong band of PDA-NPs at 1044 cm^−1^ was due to the stretching vibration of catechol hydroxyl C–O and/or C–N [32]. The presence of indole and indolequinone structures in the FTIR spectrum of the PDA sample demonstrated that dopamine was successfully polymerized to PDA-NPs [34].

**Figure 3:**
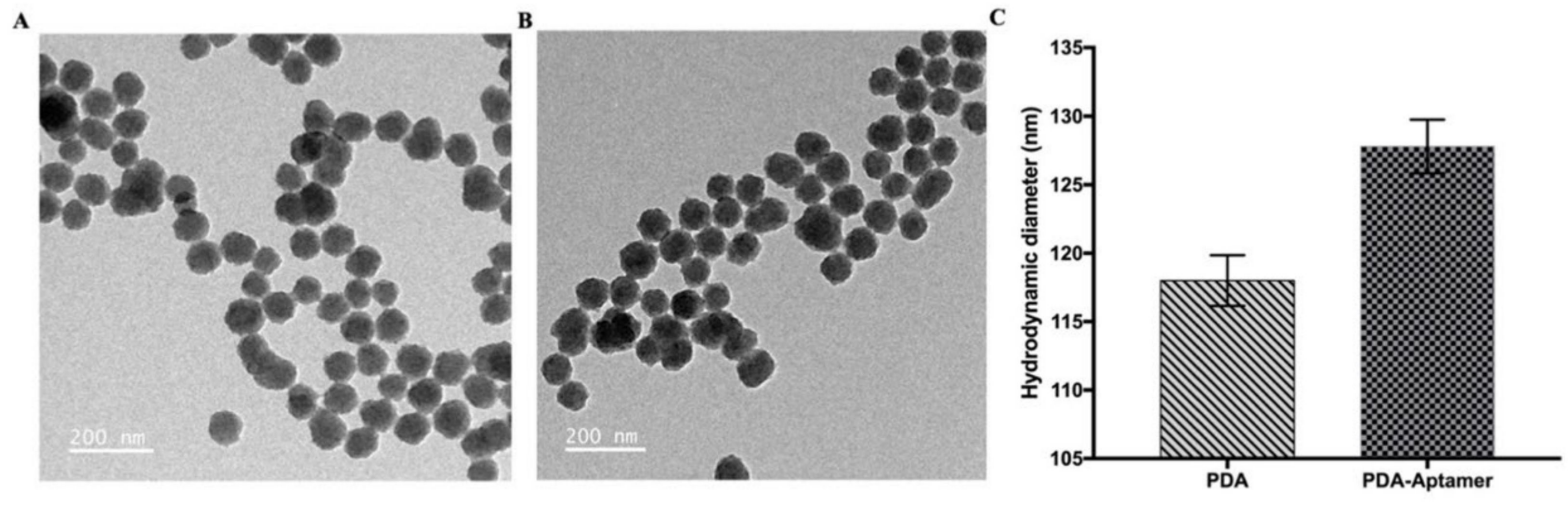
TEM images of (**A**) PDA-NPs and (**B**) PDA-aptamer NPs showing monodispersed spherical distribution. (**C**) DLS measurements of PDA-NPs and PDA-aptamer conjugates.

As shown in Figure 3A,B, under a transmission electron microscope (TEM), morphologically PDA-NPs and PDA-aptamer NPs had monodispersed spherical distribution. By dynamic light scattering (DLS) measurements, the prepared PDA-NPs were monodispersed with an average size of 118.0 ± 1.9 nm. PDA surfaces are unable to link with nucleophiles (e.g., amine and thiol groups) by Schiff base or Michael addition reactions under alkaline conditions [19]. In this study, we conjugated DNA aptamer containing an amine group at 5′ end on PDA surfaces through a covalent reaction. A reactant bearing an amine group can target the diketone or catechol groups expressed on the PDA layer via Michael addition and Schiff base reactions, which can modify the polymer surface properties [20]. To study the change of NP size after aptamer conjugation in a microcentrifuge tube, we used a similar protocol as that for the conjugation of it on SPCEs of the electrochemical sensor. After measurement by DLS, the hydrodynamic diameter of PDA aptamer NPs (127.8 ± 2.0) nm slightly increased than that of PDA-NPs, indicating successful surface modification (Figure 3C). The polydispersity index (PDI) of both PDA-NPs and PDA-aptamer NPs was within the range of 0.2 which is usually considered evidence of the homogeneity and monodispersity of NPs. After aptamer conjugation, PDA-NPs still maintained their stability. This may be due to performing conjugation under alkaline pH.

### 2.2. Characterization of Electrochemical Aptasensor

**Figure 4:**
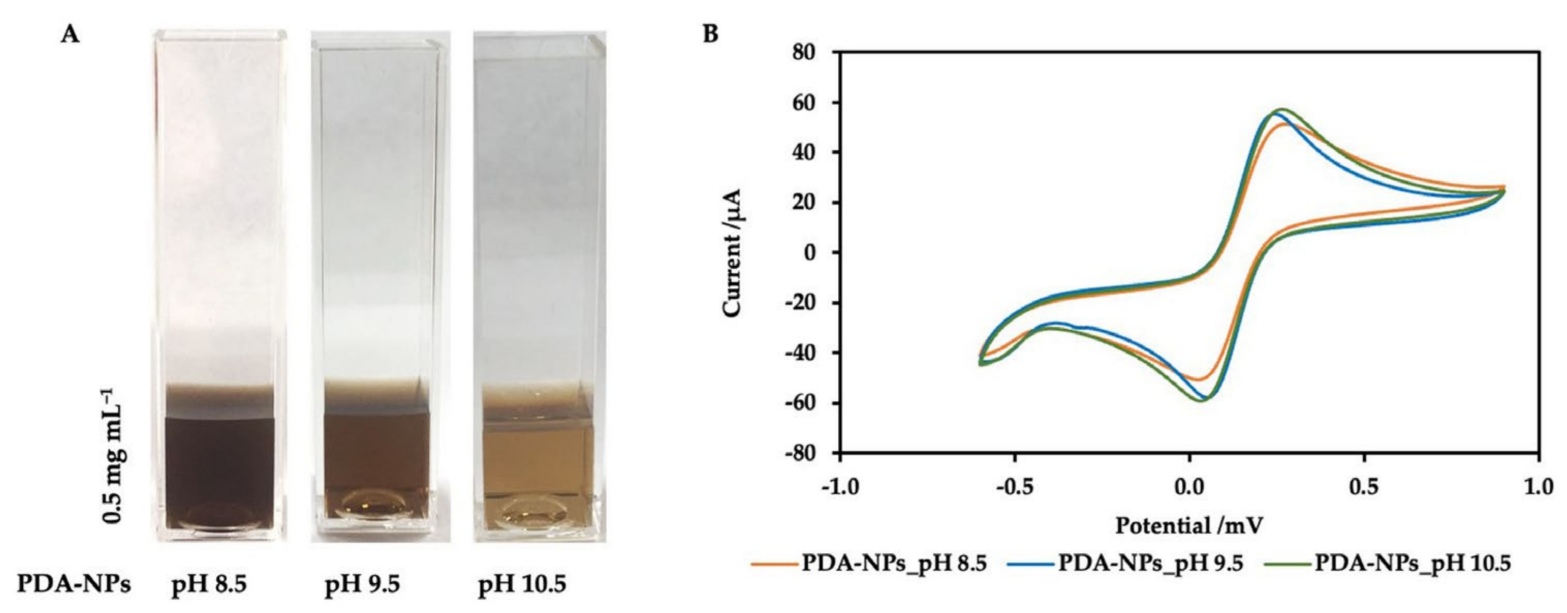
(**A**) PDA-NPs solution synthesized under different pH conditions. (**B**) Cyclic voltammograms of SPCEs modified with PDA-NPs synthesized at different pH.

PDA has now been widely applied in electrochemical biosensing because of its excellent biocompatibility, abundant functional groups (catechol, amine), facile preparation, and anti-biofouling effect [35]. In this study, PDA-NPs were produced by polymerization of its monomer dopamine in Tris buffer under highly alkaline pH conditions. Various PDA-NP sizes were synthesized by varying the pH of the Tris buffer (pH 8.5, 9.5, and 10.5), with lower basic pH leading to the gradual color changes of the solution towards dark-brown (Figure 4A) and the increase of the sizes of NPs (Table S1, Supplementary Materials). The solution of PDA-NPs was dropped onto carbon electrode surfaces to modify their surfaces. The electrochemical characterization of the developed aptasensor was characterized by cyclic voltammetry (CV) from −0.5 V to 0.9 V at a scan rate of 100 mVs^−1^ using 5 mM [Fe(CN)_6_]^3−/4−^. The electrochemical characterization by CV of the PDA-NPs after immobilization is shown in Figure 4B. PDA-NPs coated SPCEs exhibited changes in the peak current properties due to the coating, confirming surface modification with a more conductive material at pH 9.5 and 10.5, whereas PDA-NPs synthesized at pH 8.5 decreased a current signal compared to PDA synthesized at pH 9.5 and 10.5. This was caused by the formation of larger size PDA-NPs.

**Figure 5:**
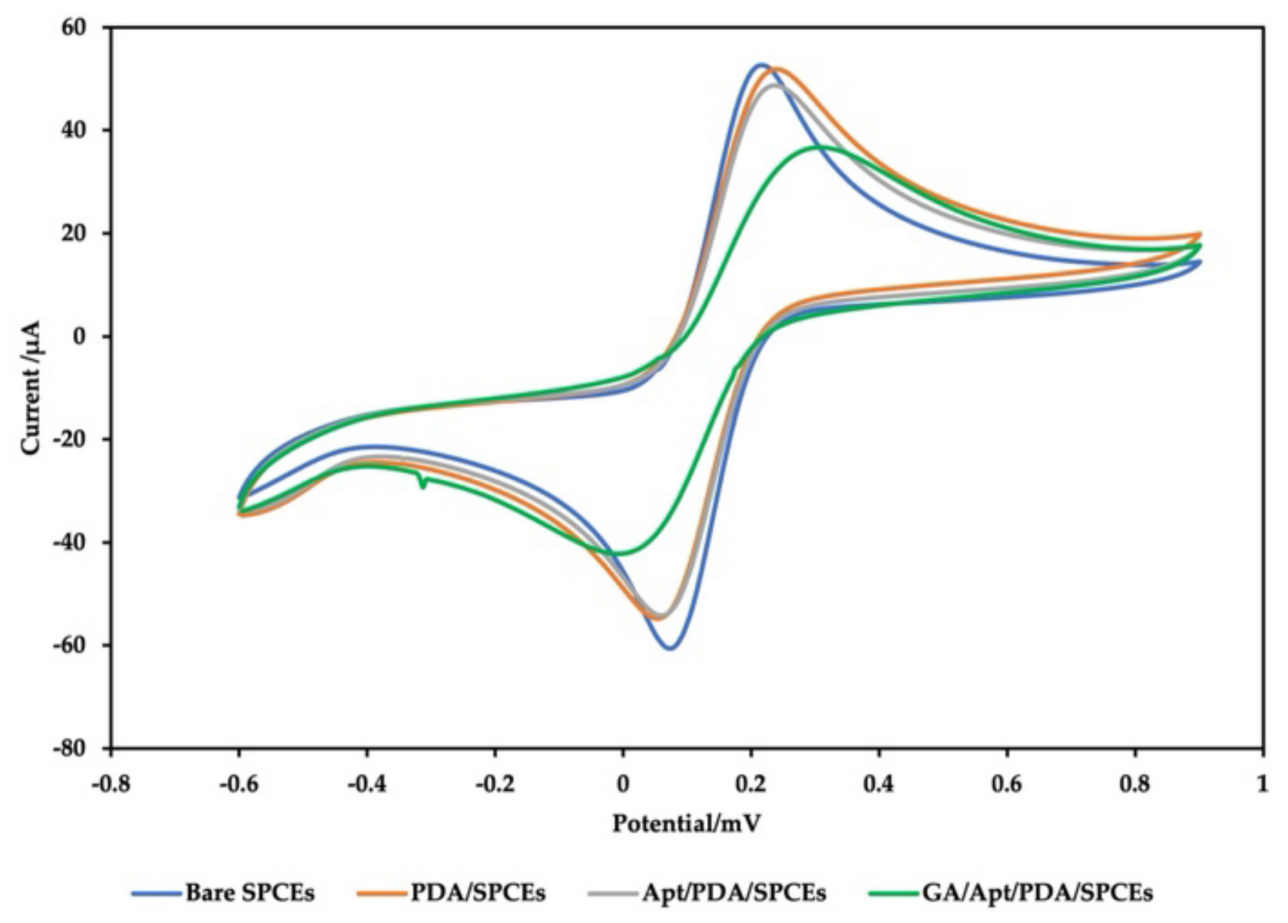
A Cyclic voltammograms of Bare SPCEs, PDA/SPCEs, Apt/PDA/SPCEs, GA/Apt/PDA/SPCEs showing successful immobilization/reaction of each layer.

**Figure 6:**
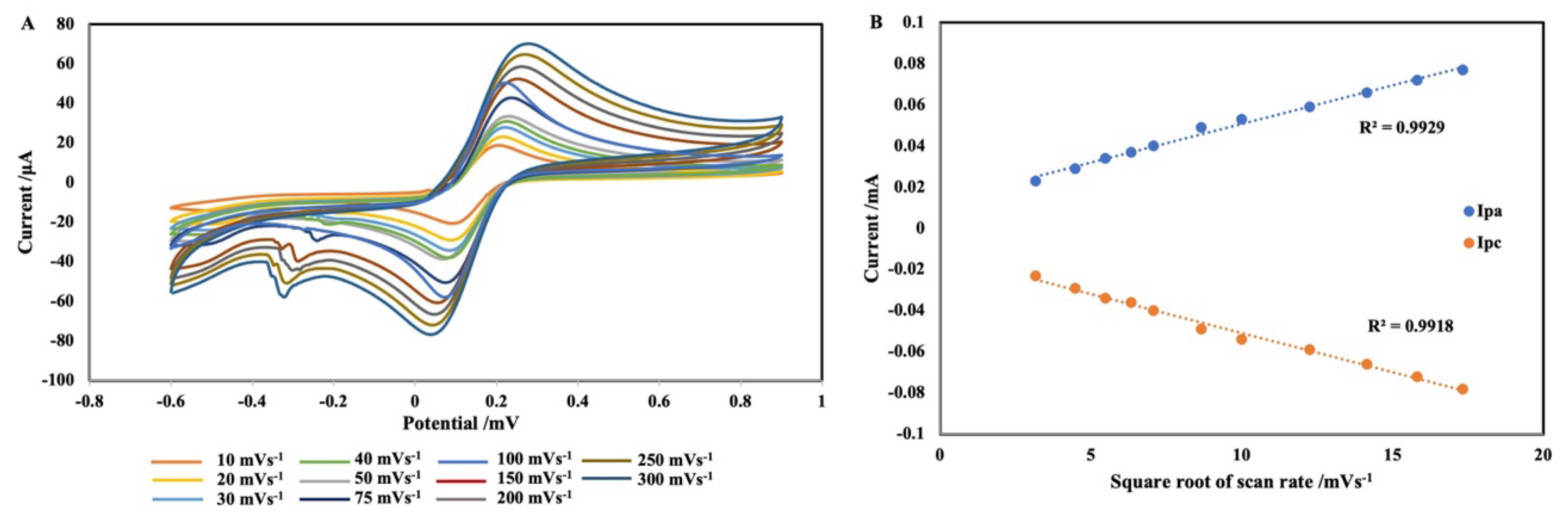
(**A**) Cyclic voltammograms of PDA/SPCEs at different scan rates from 10 to 300 mVs^−1^ using 5 mM [Fe(CN)_6_]^3−/4−^ in phosphate-buffered saline (PBS) as redox probe. (**B**) plot between anodic (Ipa) and cathodic (Ipc) peak currents vs. square root of the scan rate (mVs^−1^).

Figure 5 shows the CV of the aptasensor after each immobilization. After coating SPCEs with PDA, both anodic (Ipa) and cathodic (Ipc) peak currents were slightly decreased with an increase in the peak-to-peak separation values. This indicated the successful coating of the surface with PDA-NPs. The decrease of the redox peak currents after aptamer immobilization was caused by the negative charge on the phosphate backbone of the aptamer and its large structure. This data showed that complete immobilization of the aptamer on the PDA modified SPCEs. The peak was further reduced after target incubation because of the conformational structure change of the aptamer after specific protein binding and blocking the electron transfer process. Then, the effect of the scan rate on the voltammetric behavior of PDA/SPCEs was investigated (Figure 6A). The scan rate parameters were in the range of 10–300 mVs^−1^. Figure 6B shows a linear relationship between the square root of the scan rate and peak currents (Ipa and Ipc), with an R^2^ value of 0.9929 and 0.9918, suggesting the characteristics of the corresponding thin-layer type voltammetry [36]. The plot between the logarithms (log) of scan rates (mVs^−1^) and log peak current (Ipa and Ipc) indicated a linear relationship with the slope values of 0.49 and 0.51, respectively (data not indicated). The resulting values were in agreement with those for purely diffusion-controlled currents [37]. This implies that the PDA-modified SPCE surface was not fouled, and the electrochemical reaction was controlled by diffusion.

### 2.3. Optimization of the Experimental Conditions

The optimization of experimental parameters is crucial for getting optimal experimental results. The optimal concentration of PDA was selected from 0.5, 0.75, and 1 mg mL^−1^ PDA. However, the concentrations of PDA did not affect the electrochemical measurements (data not shown). Several conditions, including (a) measurement parameters, (b) aptamer concentration required for immobilization, (c) blocking, and (d) the reaction time with the target, were optimized. Electrochemical impedance spectrometry (EIS) is commonly used for surface characterization, batteries, corrosion studies, and semiconductors. It is also used for the biosensing of immunological reactions [38,39]. The observation of non-specific impedance changes may result in the inability to discriminate between specific and non-specific interactions [40]. Non-steady EIS signals also resulted from electrode contamination, additional voltammetric measurements, and repetitive measurements [41]. CV was used for the detection of GA concentration in this study.

#### 2.3.1. Optimization of GA Aptamer Immobilization

**Figure 7:**
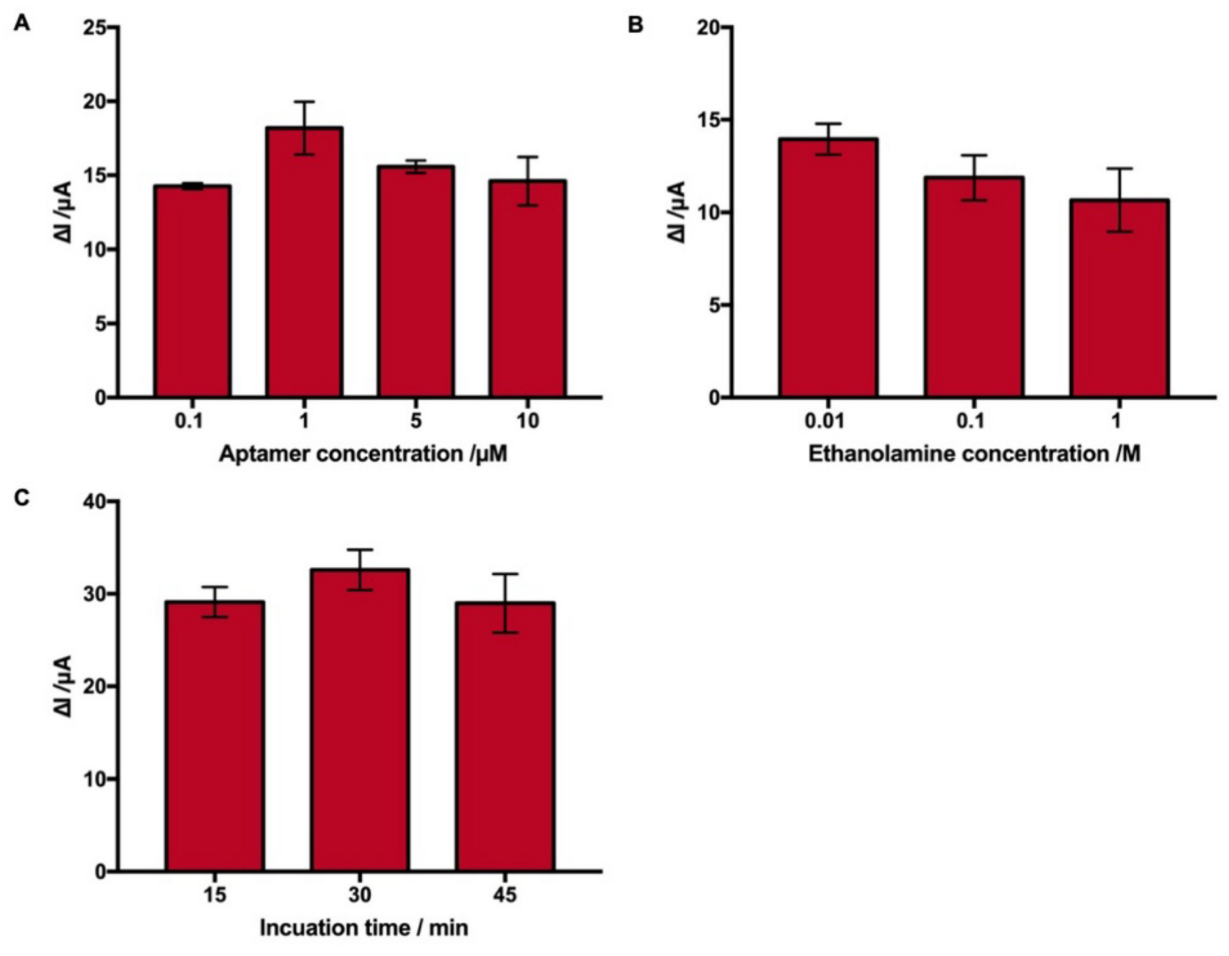
Optimization of (**A**) aptamer concentration, (**B**) concentration of blocking solution, and (**C**) reaction time with GA protein.

One of the most important steps in the development of electrochemical aptasensors is to optimize aptamer concentration. Various concentrations of aptamer (0.1–10 μM) were tested to optimize aptamer immobilization. The efficient electrochemical signal was not provided at a low concentration of the aptamer (0.1 µM). The sensitivity of the aptasensor was decreased at higher aptamer concentrations (5, 10 µM) due to dense immobilization on the electrode surface. In the context of CV current, an aptamer concentration of 1 μM produced an appropriate signal and improved sensitivity (Figure 7A). 

#### 2.3.2. Optimization of Blocking and Reaction Time

Various blocking agents such as bovine serum albumin (BSA), ethanolamine, and casein have been used. However, the similarity of the molecular structure of BSA with GA, BSA might interfere with detection. Therefore, ethanolamine was used as a blocking reagent in this study. Various concentrations of ethanolamine (0.1 M, 0.1 M, and 0.01 M) were used to determine optimal concentration. The maximum current change was observed at 0.01 M ethanolamine concentration (Figure 7B). The reaction time with the target solution was optimized ranging from 15 to 45 min. The optimal binding was achieved at 30 min with appropriate electrochemical signals (Figure 7C). 

### 2.4. Electrochemical Detection of GA

**Figure 8:**
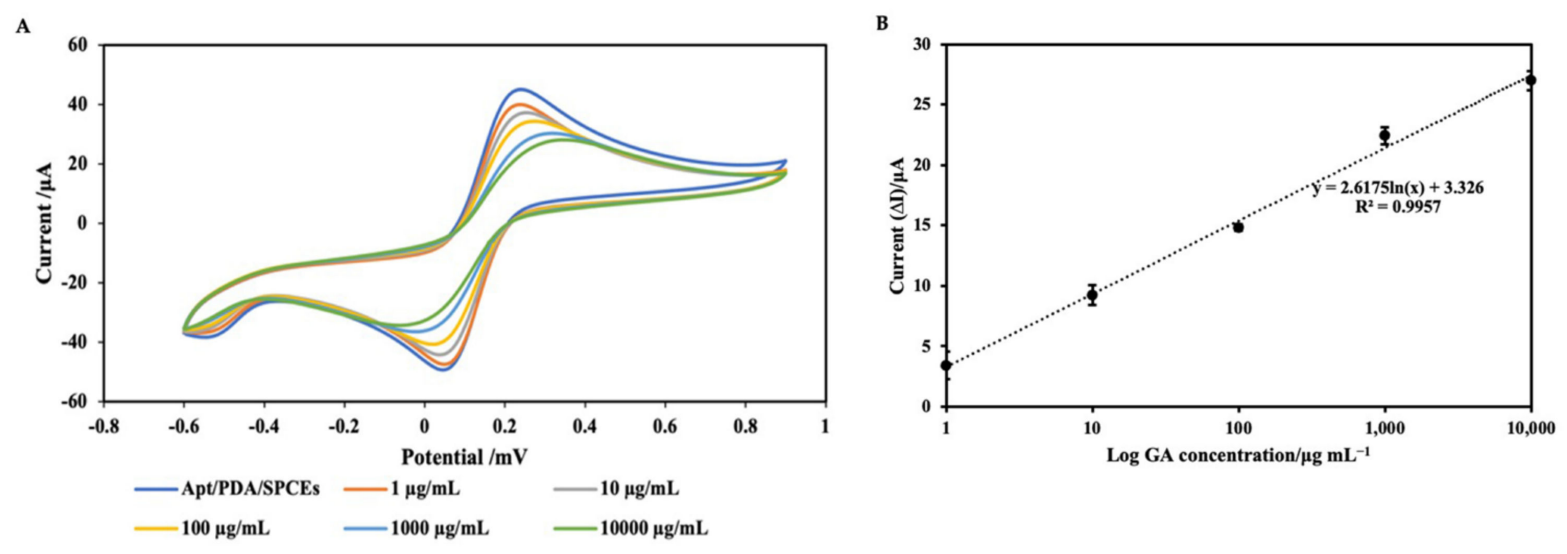
(**A**) CV response of Aptamer/PDA/SPCEs aptasensor after incubation with concentrations of 1 μg mL^−1^, 10 μg mL^−1^, 100 μg mL^−1^, 1 mg mL^−1^, and 10 mg mL^−1^. (**B**) The calibration curve for GA detection showing linear relationship between the logarithm of GA concentration 1–10,000 µg mL^−1^ and corresponding maximum current (∆I) from CV. Mean current value of each concentration was plotted from three independent experiments.

The sensitivity of the developed aptasensor was tested against various concentrations of GA (from 1 to 10,000 μg mL^−1^) under optimal conditions. The aptamer immobilized on PDA-NPs/SPCEs provided a relatively significant current exhibiting a free and steady assembly on the electrode surface. When the aptamer changed conformational structure upon binding to the target, the captured GA-aptamer complex on the sensor surface acted as a kinetic barrier, inhibiting the electron transfer reaction, and thus lowering the peak current. The decrease of the CV oxidative peak current was observed corresponding to the increase in GA concentration (Figure 8A). Electrochemical measurements were carried out before and after target binding with the aptamer. The peak current changes were calculated as the following equation: ∆I = I_0_ − I_1_, where ∆I is the peak current change, and I_0_ and I_1_ are the peak current before and after incubation with the target protein, respectively. The efficient binding of GA by the aptasensor was indicated by a decrease in the maximum peak current. Figure 8B shows a standard curve plotted between the logarithm of the GA concentration and the peak current change (µA). The peak current changes increased linearly with log GA concentrations (I = 2.6166 ln(c) + 3.326), with a correlation coefficient (R^2^) of 0.9957 for GA detection over a range of 1–10,000 µg mL^−1^. The limit of detection (LOD) of GA was calculated from the equation; LOD = 3σ/S, where σ is the standard deviation of the average measurement of the lowest concentration of GA and S is the slope of the regression line. The calculated LOD value of the developed aptasensor was 0.40 µg mL^−1,^ which is sensitive enough to detect GA concentrations due to the normal range of serum GA being 0.2–7 mg mL^−1^ [42]. The aptasensor developed here is highly sensitive enough with a wide linearity range from 1 to 1 × 10^4^ µg mL^−1^. The calculated LOD in this study was compared to those of the previous studies (Table 1). Bunyarataphan et al. and Aye et al. reported the electrochemical aptasensors for GA detection with the low LOD of 3 ng mL^−1^ and 31 ng mL^−1^, respectively [7,16]. However, the former sensor fabrication process involved prolonged incubation for streptavidin to be firmly immobilized on the electrode surface, which was resolved in this study with reduced fabrication time. The graphene oxide (GO)-modified aptasensor presented simple and sensitive biosensing functions [16]. However, the interference with high human serum albumin (HSA) concentration limited the selectivity of the aptasensor which was surpassed in our study. A detailed comparison of the selectivity between these methods can be found in the following section. In the sensing systems, the binding affinity of aptamer and GA, the physical properties of the PDA-NPs, and the optimal conditions of aptamer concentration, blocking concentration, and reaction time, all have a significant impact on the detection sensitivity and specificity. In this study, PDA-NPs functionalized electrochemical aptasensor showed a linear response over a larger range (0.001–10 mg mL^−1^) of other approaches. Therefore, the aptasensor developed in this study can confidently be applied for sensitive detection of both low and high GA levels of clinical samples.

### 2.5. Selectivitiy and Reproducibility of the Electrochemical Aptasensor

**Figure 9:**
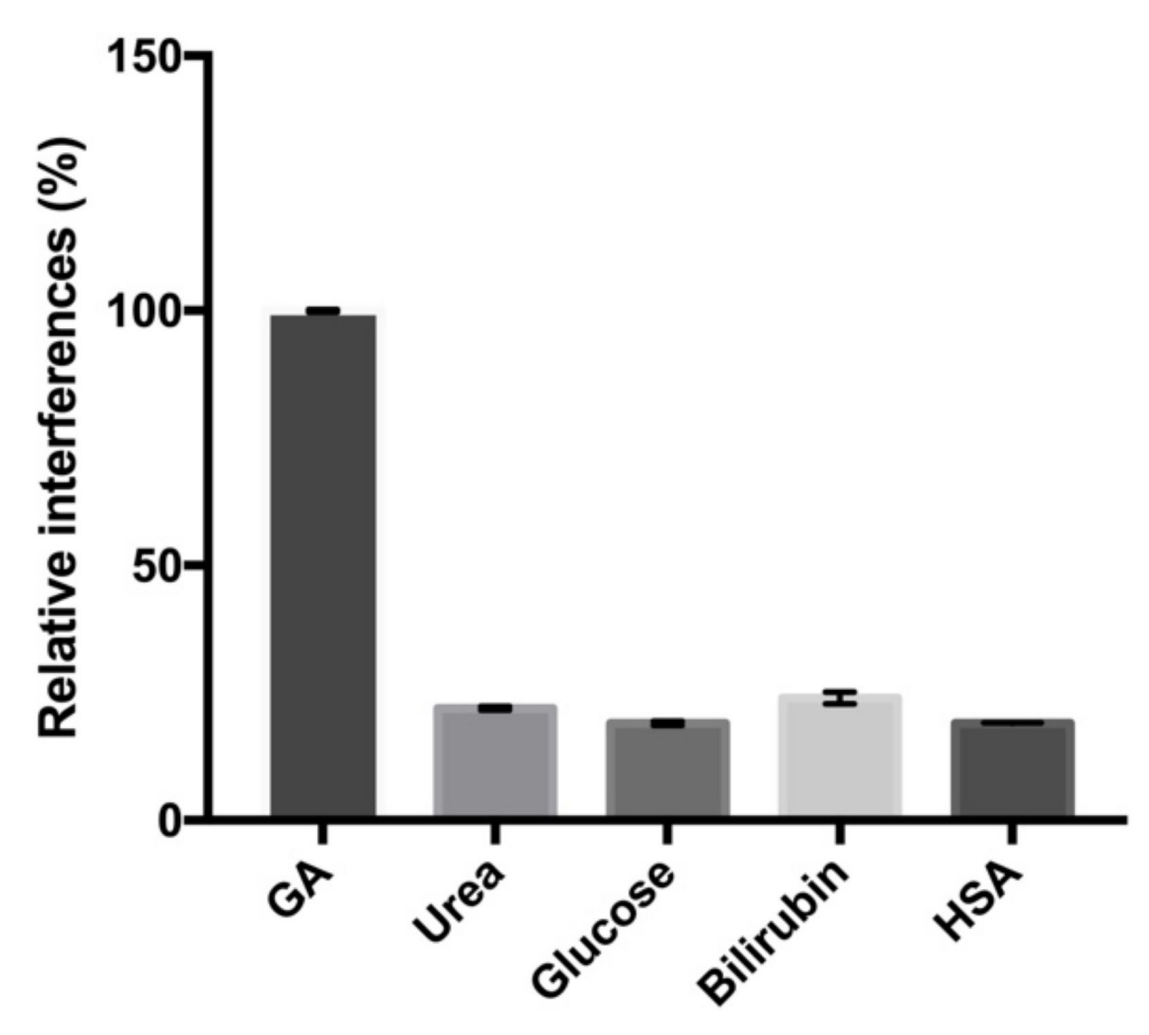
Specificity of electrochemical aptasensor to GA (1 mg mL^−1^) compared with other biomaterials urea (2.5 mg mL^−1^), glucose (125 mg dL^−1^), bilirubin (2 mg dL−1), and HSA (100 µg mL^−1^).

To verify that the current changes were in fact caused by specific interaction between the aptamer and GA, the specificity of the aptasensor was evaluated using common interfering substances such as urea (2.5 mg mL^−1^), glucose (125 mg dL^−1^), bilirubin (2 mg dL^−1^) and HSA (100 µg mL^−1^). Apparently, as shown in Figure 9 the largest current change was observed with GA protein. In contrast, only small current changes (22 ± 0.39%, 19 ± 0.53%, 24 ± 1.14%, 19 ± 0.09%) were observed with urea, glucose, bilirubin, and HSA, respectively. The concentrations of the interfering substances (except for HSA) used here were higher than their normal level in the normal human serum. Cross-reactivity with HSA is still a challenge for most GA biosensors as these two molecules possess a relatively similar molecular structure [7,9]. The present results indicate that the aptasensor developed here exhibited high specificity for the detection of GA without any labeling.

Five independent experiments against three different concentrations of GA at 0, 0.1, and 10 mg mL^−1^ were carried out to test the reproducibility of the aptasensor detection. The relative standard deviations (RSDs) obtained were 8.28%, 1.77%, and 2.82%, respectively (Table 2). The RSD is lower than the acceptable value (less than 10%) [48] proving that the aptasensor can generate highly reproducible electrochemical signals and good precision. Moreover, the consistency of the electrode fabrication process was perceived. The stepwise electrode modification strengthened the reproducibility of the aptasensor. The relative interferences with HSA and reproducibility of the developed electrochemical aptasensor were compared to other methods in Table 3.

### 2.6. Spike Recovery Assay

**Figure 10:**
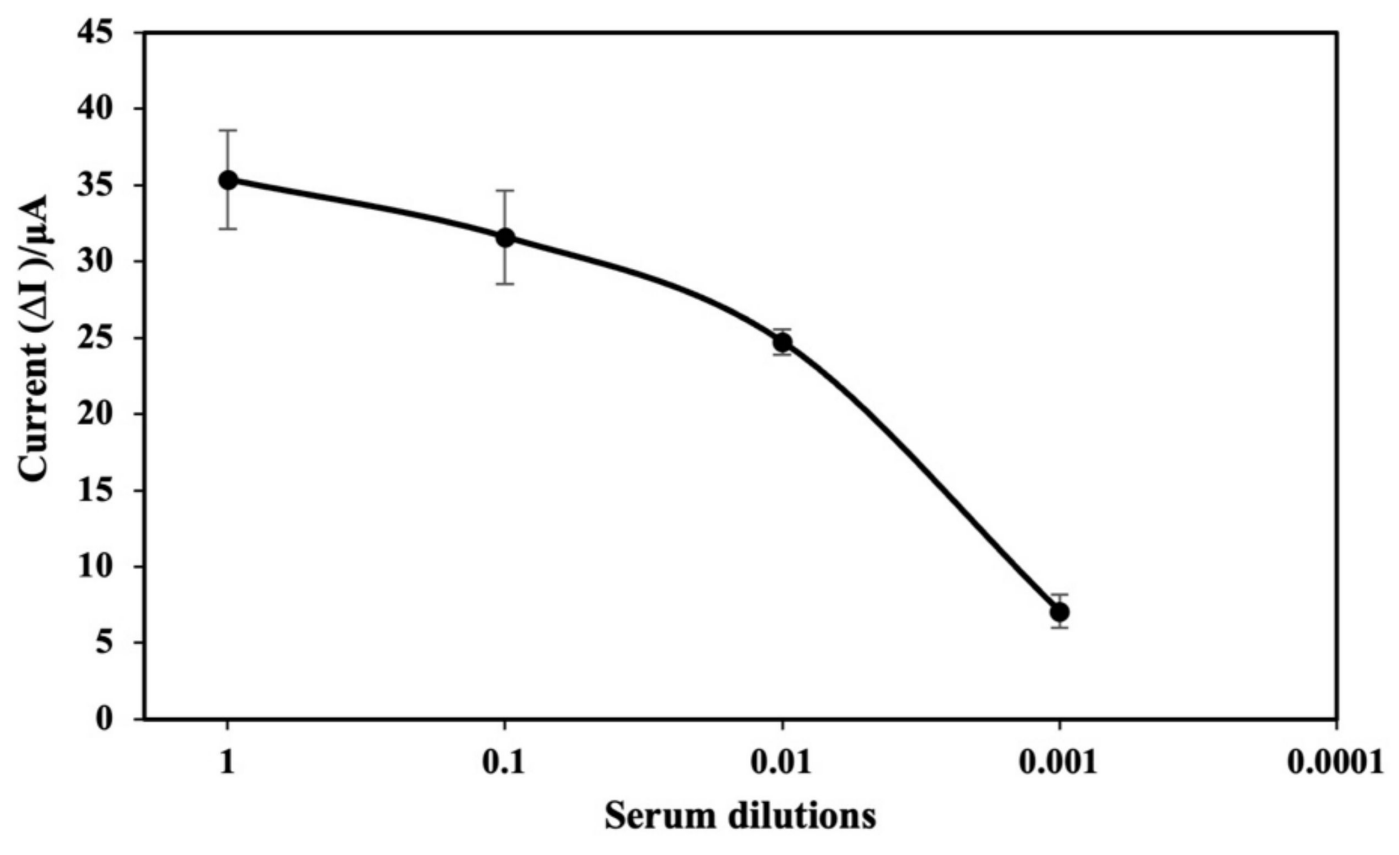
Evaluation of optimal serum dilution for spike recovery assay.

The accuracy of the GA detection of the electrochemical aptasensor developed in this study was evaluated using a GA-spiked human serum. Prior to spiking with GA, the serum was diluted from 1:1 to 1:1000 with PBS (1X, pH 7.4) to investigate the optimal serum dilution. The current changes were decreased in parallel with serum (Figure 10). Since relatively small interference with small current change was observed at 1:1000 dilution, this dilution was chosen to reduce the interference from other serum proteins.

To demonstrate the applicability and feasibility of the fabricated aptasensor for clinical samples, two different concentrations (47.60 and 238.10 µg mL^−1^) of GA solutions were spiked into the diluted serum. The recovery rates of low and high GA concentrations were 104% and 90%, respectively, as shown in Table 4, which were within the acceptable range with RSD values of 6.10% and 2.80%, respectively. The data showed that the aptasensor developed here can detect GA levels accurately. Therefore, the developed aptasensor is observed to be reliable and can be used as a potential tool for the detection of GA in real clinical samples. However, further validation experiment including the comparison of the developed biosensor and a reference method for real sample analysis is required prior to implementation in the clinical laboratory.

## 3. Materials and Methods

### 3.1. Reagents and Materials

The GA binding aptamer with a 33-nucleotide sequence of 5′-GG TGG CTG GAG GGG GCG CGA ACG TTT TTT TTT T–3′ [49] was modified by an amino group (NH_2_) at 5′ end of the aptamer sequence (Integrated DNA Technologies Pte. Ltd., Singapore). The aptamer was reconstituted using Tris-EDTA (TE) buffer (1X, pH 8.2). Dopamine hydrochloride, GA, and ethanolamine were purchased from Sigma-Aldrich (Singapore). Disposable screen-printed carbon electrodes (SPCEs, 30 × 12.5 mm) were purchased from Quasense, Thailand. As a redox indicator, potassium ferricyanide (K_3_[Fe(CN)_6_], 5 mM) together with 0.1 M potassium chloride (KCl) in phosphate-buffered saline (PBS) (1X, pH 7.4). All reagents were prepared in ultrapure deionized water (DI).

### 3.2. Instruments

The electrochemical detection system was completed by the transducer (the electrode sensor) and the detector. The transducer was a disposable SPCE with a three-electrode system including a 3-mm working carbon electrode, a carbon counter electrode, and a silver/silver chloride reference electrode. The PalmSens4 potentiostat with PS Trace 5.8 software (PalmSens BV Co., Ltd., Houten, The Netherlands) was used for all electrochemical experiments. The characterization of PDA-NPs was carried out by an Eppendorf BioSpectrometer^®^ fluorescence (Hamburg, Germany), a Bruker TENSOR II ATR-FTIR spectrometer (Bruker, Germany), a transmission electron microscope (TEM, FEI, TECNAI G2 20, Nieuw-Vennep, The Netherlands), a scanning electron microscope (SEM, Jeol, JSM-IT200 InTouchScope™, Tokyo, Japan) and a dynamic light scattering (DLS, Malvern, UK).

### 3.3. Synthesis of PDA-NPs and In Vitro Conjugation of GA Aptamer on PDA-NPs

PDA-NPs were synthesized by simply dissolving 50 mg of dopamine hydrochloride into 100 mL of 10 mM Tris buffer (pH 8.5, 9.5, and 10.5). The suspension was stirred at 180 rpm for 20 h. The solution was then centrifuged at 16,100× *g* for 5 min at room temperature. PDA-NPs were washed twice with 1 mL of Tris buffer. The supernatant was discarded, and the remaining pellets were collected. The PDA-aptamer complex was prepared by mixing equal volumes of synthesized PDA-NPs and GA-specific aptamer. Aptamer conjugation was done 45 min after incubation.

### 3.4. Fabrication of the Electrochemical Aptasensor

The working carbon electrodes were coated with seven microliters of the PDA-NPs solution and dried in the oven at 50 °C for 30 min. To remove excess PDA-NPs, DI was used to thoroughly clean the electrodes. Then, 7 µL of 1 µM GA aptamer were immobilized and stabilized on the working electrode for 30 min to allow complete binding between the amino groups of the aptamers on the PDA surfaces. PBS (1X, pH 7.4) was used to completely wash the unbound aptamers. The surfaces were blocked with 0.1 M ethanolamine to reduce non-specific binding. After blocking for 15 min, the SPCEs were washed with PBS again. The layer-by-layer aptasensor was applied for the detection of GA in this study.

### 3.5. Electrochemical Analysis

**Scheme 1:**
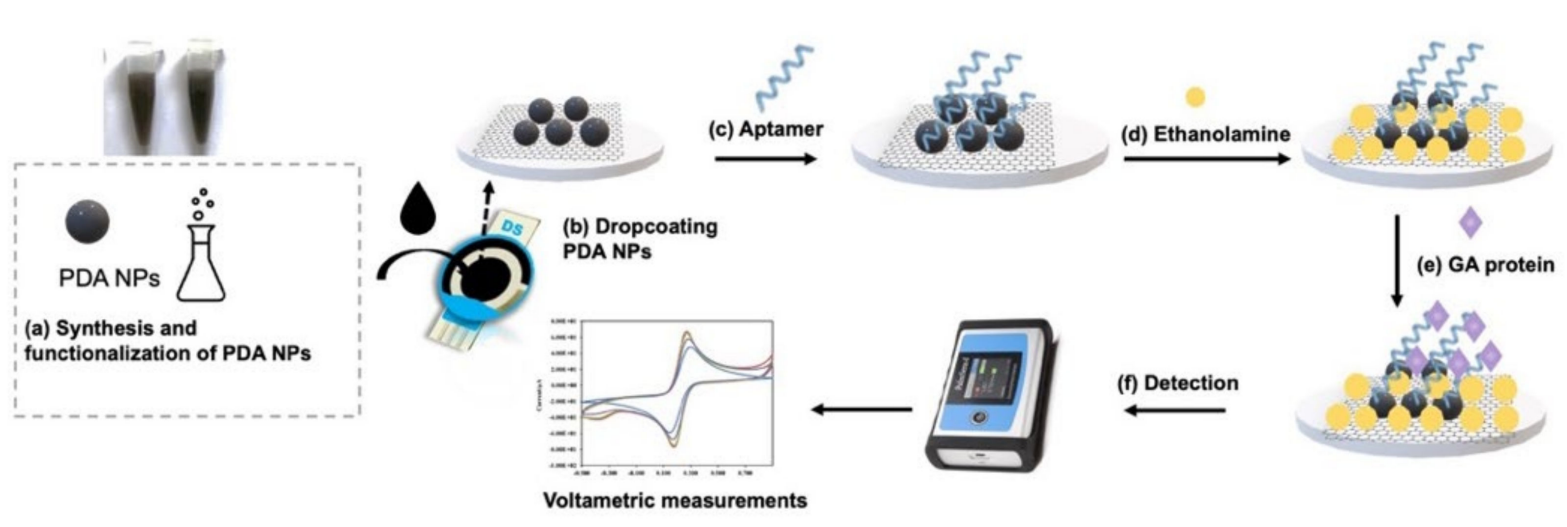
Schematic diagram of the proposed PDA-NPs functionalized electrochemical aptasensor for GA protein detection (**a**) Synthesis and functionalization of PDA-NPs (**b**) Drop-coating PDA-NPs onto SPCEs (**c**) Immobilization of aptamer (**d**) Blocking with ethanolamine (**e**) Incubation with GA and (**f**) Voltametric measurements using 5 mM [Fe(CN)_6_]^3−/4−^ as a redox indicator.

The produced aptasensors were incubated with various GA concentrations for 30 min. Then, electrode surfaces were rinsed with PBS and 130 µL of 5 mM [Fe(CN)_6_]^3−/4−^ was added until the surfaces were immersed. For the characterization of the fabrication process, CV and EIS analyses were carried out after each immobilization procedure. Both electrode characterization and GA detection were measured by CV with a potential of −0.5 V to 0.9 V at a scan rate of 100 mVs^−1^ using a redox indicator. The impedance spectra were measured using the open circuit potential at the frequency range of 100 mHz to 100 kHz. The flow diagram of the proposed electrochemical aptasensor is illustrated in Scheme 1.

### 3.6. Real Sample Analysis

After optimal conditions were obtained, the capability of the proposed electrochemical biosensor was investigated in real samples. Serum samples were spiked with GA at final concentrations of 47.60 and 238.10 µg mL^−1^ to test the clinical performance of the proposed biosensor. Known concentrations of GA spiked into diluted serum samples were determined by employing the electrochemical measurements that were described in the previous section.

## 4. Conclusions

PDA is quite a promising sensor material for a variety of analyses. In particular, it has been extensively applied for designing biochips for biomarker detection. In this study, we used functional PDA-NPs as a coating material on SPCE surfaces to create an electrochemical GA detector under a new concept, which has the potential to monitor glycemic control in DM patients. Here, we designed the sensing system to be convenient with a simple and facile fabrication process and label-free GA detection. The PDA aptasensor developed here showed excellent sensitivity, selectivity, and a low detection limit of 0.40 µg mL^−1^ for GA detection. This proposed technique shows its potential application in serum/plasma GA measurement to improve the screening and management of diabetic patients in the future. It can be also applied to new PDA-based nanoconstructs to modify the electrode surfaces for a variety of biomedical applications.

## Figures and Tables

**Table 1:** Comparison of limit of detection using various biosensors for the detection of serum glycated albumin.

No.	Techniques	Limit of Detection (LOD)	Linear Response Range	References
1.	Nanozyme-based electrochemical immunoassay	3.8 µg mL^−1^	5 µg mL^−1^ to 10 mg mL^−1^	[43]
2.	rGO/AuNPs based electrochemical aptasensor	0.07 μg mL^−1^	2–10 μg mL^−1^	[8]
3	Electrochemical-based aptasensor	0.003 μg mL^−1^	0.002–16 mg mL^−1^	[7]
4.	Cu_2_O–rGO nanozyme-based electrochemical sensor	0.007 μg mL^−1^	0.02–1500 μg mL^−1^	[44]
5.	Paper-based device with boronic acid-derived agarose beads	7.1 μg mL^−1^	10 μg mL^−1^ to 10 mg mL^−1^	[45]
6.	Colorimetric immunoassay using Prussian blue nanoparticles	7.32 μg mL^−1^	0.01–2.0 mg mL^−1^	[46]
7.	Enzymatic assay-based sensor	0.36 µg mL^−1^	0–0.6 mg mL^−1^	[42]
8.	rGO-based aptasensor	16.40 µg mL^−1^	0–125 µg mL^−1^	[47]
9.	GO-fucntionalized electrochemical aptasensor	0.031 µg mL^−1^	0.001–10 mg mL^−1^	[16]
10.	PDA-NPs functionalized electrochemical aptasensor	0.40 µg mL^−1^	0.001–10 mg mL^−1^	This study

**Table 2:** Reproducibility of the aptasensor detection for various concentrations of GA (0, 1 and 10 mg mL^−1^).

GA Concentration (mg mL^−1^)	Peak Current (µA)	Relative Standard Deviation (%) (*n* = 5)
**0.00**	3.91 ± 0.32	8.28%
**0.10**	14.83 ± 0.26	1.77%
**10.00**	26.98 ± 0.76	2.82%

**Table 3:** Comparison of selectivity and reproducibility of the developed electrochemical sensor with other reported sensors.

No.	Biosensors	Relative Interferences with HSA (%)	Reproducibility (CV%)	References
1.	Electrochemical-based aptasensor	29 ± 3%	6.50%	[7]
2.	Immobilization free electrochemical sensor	52.91 ± 1.46%	9.88%	[9]
3.	GO-functionalized electrochemical aptasensor	26.2 ± 0.2%	2.50%	[16]
4.	PDA-NPs functionalized electrochemical aptasensor	19 ± 0.09%	2.82%	This study

**Table 4:** Recovery assay of GA in human serum using the developed aptasensor.

GA concentration Spiked in Serum (µg mL^−1^)	Measured Concentration (µg mL ^−1^)	Recovery (%)	RSD ^a^ (%) (*n* = 3)
**47.60**	53.1 ± 0.79	104	6.10
**238.10**	217 ± 0.56	90	2.80

^a^ The relative standard deviations.

## Data Availability

Not applicable.

## Supplementary Materials

The following supporting information can be downloaded at: https://www.mdpi.com/article/10.3390/ijms232213699/s1, Table S1: Hydrodynamic diameter and polydispersity index (PDI) of PDA-NPs synthesized at different pH.
