# Supplementary material for: Polydopamine Nanoparticles Functionalized Electrochemical DNA Aptasensor for Serum Glycated Albumin Detection
Source: Int J Mol Sci. 2022 Nov 8;23(22):13699. doi: 10.3390/ijms232213699 (PMC9690818; doi:10.3390/ijms232213699)
Supplement: Supplementary file 1 [file ijms-23-13699-s001.zip › ijms-1981821-supplementary.pdf]

Polydopamine Nanoparticles Functionalized Electrochemical DNA Aptasensor for Serum Glycated Albumin Detection

Pornsuda Maraming [1], Nang Noon Shean Aye [1], Patcharee Boonsiri [2], Sakda Daduang [3] and Onanong Buhome [4] and Jureerut Daduang [1,*]

[1] Centre for Research and Development of Medical Diagnostic Laboratories, Faculty of Associated Medical Sciences, Khon Kaen University, Khon Kaen 40002, Thailand
[2] Department of Biochemistry, Faculty of Medicine, Khon Kaen University, Khon Kaen 40002, Thailand
[3] Division of Pharmacognosy and Toxicology, Faculty of Pharmaceutical Sciences, Khon Kaen University, Khon Kaen 40002, Thailand
[4] Department of Medical Technology, Faculty of Allied Health Sciences, Nakhon Ratchasima College, Nakhon Ratchasima 30000, Thailand
* Correspondence: Correspondence: jurpoo@kku.ac.th

Table S1. Hydrodynamic diameter and polydispersity index (PDI) of PDA-NPs synthesized at different pH.

	**The synthesized PDA-NPs under different pH Tris buffer**	**Hydrodynamic diameter (*D*H in nm)**	**PDI**
**1.**	pH 8.5	286.1 ± 1.701	0.258
**2.**	pH 9.5	142.2 ± 1.595	0.123
**3.**	pH 10.5	118.0 ± 1.912	0.126
